# Supplementary material for: Experimental study on the mechanism of surface damage induced by abrasive particles under heavy-load line-contact sliding-rolling conditions
Source: PLoS One. 2025 Nov 13;20(11):e0335348. doi: 10.1371/journal.pone.0335348 (PMC12614513; doi:10.1371/journal.pone.0335348)
Supplement: Attachment — Submitted filename: Response to Reviewers.docx [file pone.0335348.s003.docx]

1. **Requirement 1**: Please ensure that your manuscript meets PLOS ONE's style requirements, including those for file naming.

**Response**：We have revised this manuscript according to the formatting requirements of PLOS ONE. The modifications are marked in red and saved in the "Revised Manuscript with Track Changes" document.

2. **Requirement 2** : Please note that PLOS ONE has specific guidelines on code sharing for submissions in which author-generated code underpins the findings in the manuscript. In these cases, we expect all author-generated code to be made available without restrictions upon publication of the work.

**Response：**The experiments in this study were conducted on a JPM-1 friction and wear testing machine, with experimental data recorded in real time. The experimental data can be found in the attached file "Data set. excel".

3.**Requirement 3**: Thank you for stating the following financial disclosure: “National Natural Science Foundation of China (51875154)；Key Project of Natural Science Research in Colleges and Universities of Anhui Province (2024AH050503)；Anhui Provincial Key Research and Development Program (202004a05020057)”Please state what role the funders took in the study.

**Response：**The role of the funder has been stated in the cover letter and highlighted in red. Please change the online submission form on our behalf. Thank you.

4. **Requirement 4**: About funding information

**Response**: Any funding-related text has been removed from the manuscript. The updated funding statement has been provided in the cover letter. Please change the online submission form on our behalf. Thank you.

5. **Requirement 5:** Please confirm at this time whether or not your submission contains all raw data required to replicate the results of your study. Authors must share the “minimal data set” for their submission.

**Response**: Our submission includes all the raw data necessary to replicate the findings of our study. The "minimal data set" has been uploaded as an attachment, named "Data set".

6. **Requirement 6:** If the reviewer comments include a recommendation to cite specific previously published works, please review and evaluate these publications to determine whether they are relevant and should be cited. There is no requirement to cite these works unless the editor has indicated otherwise.

**Response**: The reviewer did not make any suggestions in this regard.

7. **Requirement 7:** Please review your reference list to ensure that it is complete and correct.

**Response:** We ensure that the references are complete and accurate, with no citations to retracted papers.
